# Calcifying Fibrous Tumor Complicated by Empyema

**DOI:** 10.7759/cureus.8729

**Published:** 2020-06-20

**Authors:** Nikita Jain, Anchit Bharat, Nadiia Marenych, Michal Kubiak, Karam Khaddour

**Affiliations:** 1 Internal Medicine, Chicago Medical School, Rosalind Franklin University of Medicine and Science, McHenry, USA; 2 Internal Medicine, Indiana University Health Ball Memorial Hospital, Muncie, USA

**Keywords:** calcifying fibrous tumor, pseudotumor, lung mass, empyema

## Abstract

Calcifying fibrous tumor (CFT) is a rare, benign proliferation of fibroblasts and inflammatory cells that is non-invasive and usually arises in deep tissue structures. Due to its overall paucity, no accurate incidence has been reported yet. We understand this disease via a handful of case studies published in the medical literature, first of them being from 1988. Earlier known as 'pseudotumor', it was recently given its name due to the potential of recurrence and multifocal involvement. We describe the case of a 43-year-old Hispanic male who presented with a large symptomatic pleural-based mass which turned out to be CFT and was later complicated by empyema. Our aim is to increase awareness about this rare disease and throw light upon its benign nature, despite an alarming and suspicious appearance on imaging. Due to the large size of our patient’s mass (largest reported yet), he needed extensive chest wall reconstruction, leading to complications requiring additional invasive procedures. This underscores the importance of early diagnosis and treatment which can reduce the need for aggressive surgical manipulation and avoid postoperative complications, thereby providing high-value care. Treating physicians should be mindful of this, in order to prompt early recognition to ensure effective patient care.

## Introduction

Calcifying fibrous tumor (CFT) is a rare, benign proliferation of fibroblasts comprising of dense collagen, lymphoplasmacytic infiltrate, and psammomatous or dystrophic calcifications [1]. An accurate incidence is difficult to calculate due to its rarity. Only a few cases have been described in the medical literature with the first report in 1988 in the pediatric population [2]. They tend to arise from deep tissue structures, as multiple or small solitary lesions, and are usually non-invasive. Most commonly involved sites are stomach and small intestine, although any site can be involved. Pleural-based disease has been reported less often, with roughly less than 20 cases documented to date. We describe this rare disease in a 43-year-old male who presented with the largest pleural-based CFT reported to date, which seemingly involved the ribs and adjacent lung, and was later complicated by empyema.

## Case presentation

**Figure 1:**
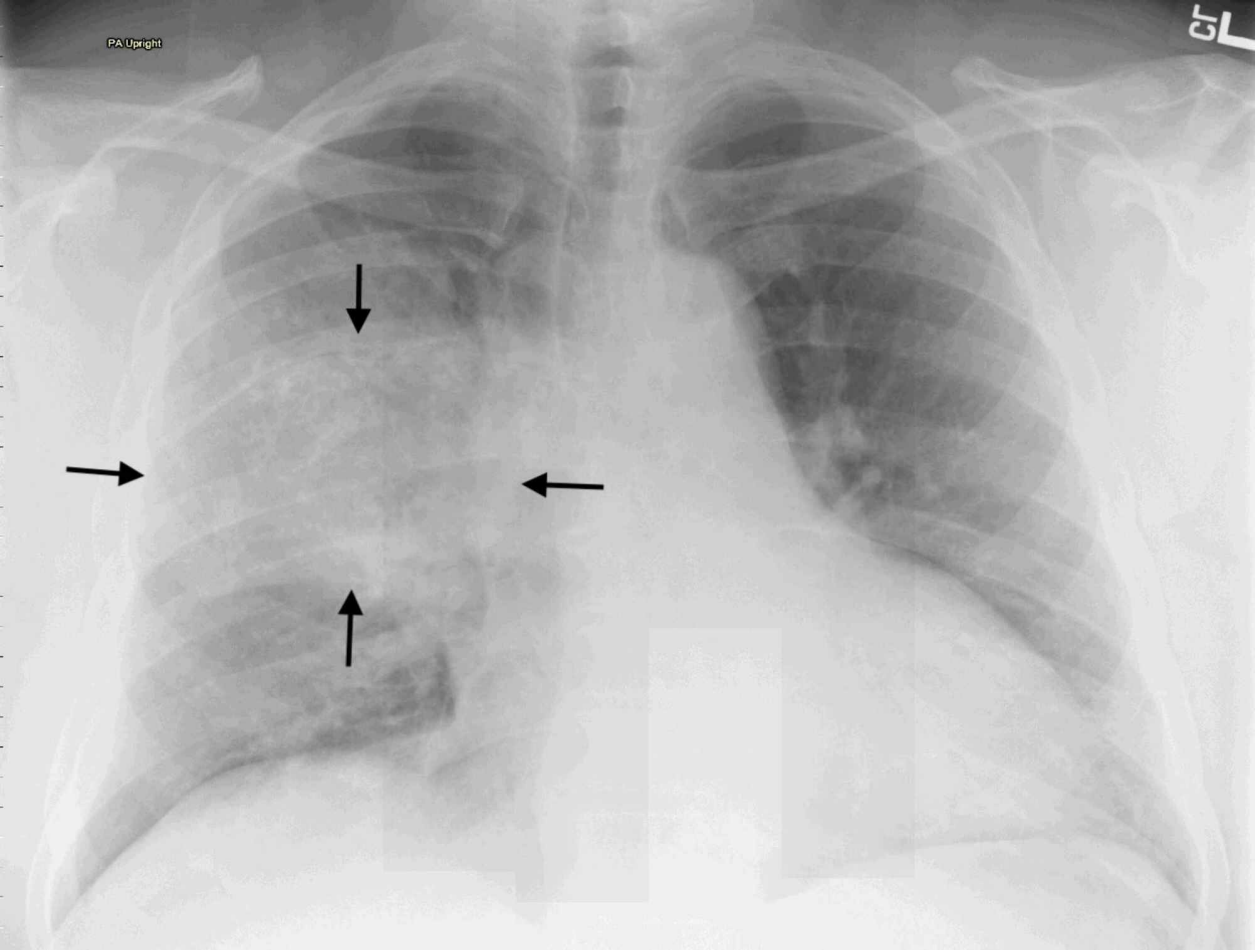
Chest x-ray showing a large shadow (arrows) extending across the right upper and middle lobes of the lung.

A 43-year-old Hispanic male with active smoking history presented with new-onset right-sided scapular pain. Past medical history was negative for cardiopulmonary disease. Physical examination revealed expiratory and inspiratory wheezing over the right upper lung zone. Routine laboratory studies were unremarkable. Chest x-ray (CXR) showed a right middle lobe pleural-based mass with stippled calcification, measuring 12 x 7 cm (Figure 1).

**Figure 2:**
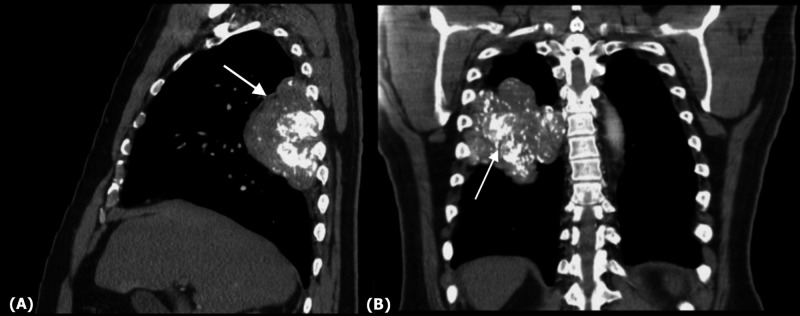
CT of the chest with a pleural-based mass occupying the posterior right lung. (A) The mass is seen abutting the adjoining ribs on a sagittal view (arrow). (B) Coronal section showing scattered calcifications within the mass (arrow) along with compression of the nearby lung.

This was followed by CT of the chest showing a large irregularly shaped mass in the posterior right hemithorax, which appeared to involve the middle and lower lobes of the lung, abutting the intercostal space between ribs 5 through 8 (Figure 2A). Additionally, significant intrinsic calcifications were noted within the mass (Figure 2B).

**Figure 3:**
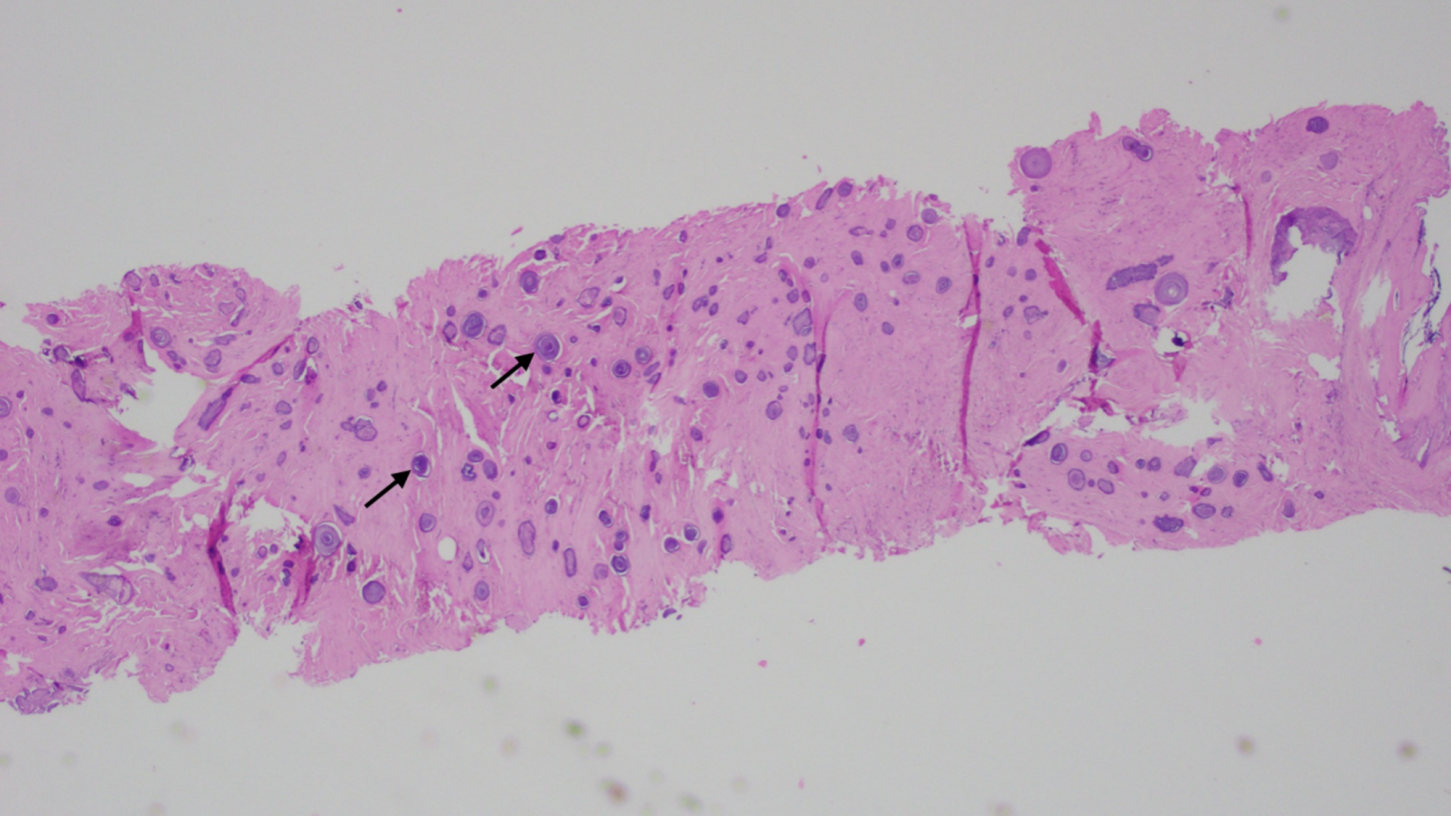
H&E stain under low power showing a focus of CFT with dense fibrous material along with calcium deposits called psammoma bodies (arrow). H&E, hematoxylin and eosin; CFT, calcifying fibrous tumor.

**Figure 4:**
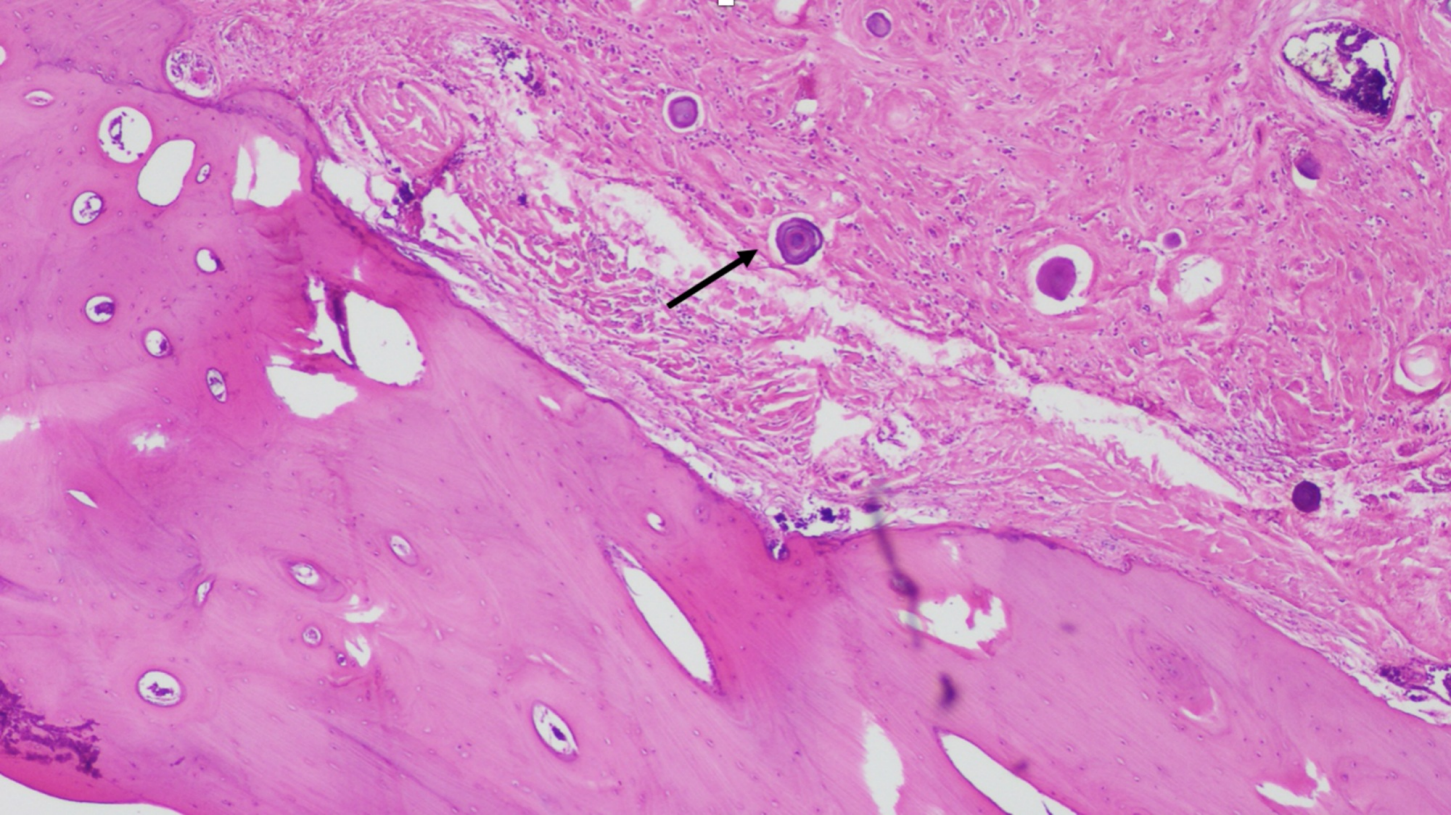
H&E stain showing the tumor in close proximity to the adjoining rib. Psammoma bodies better visualized under high power (arrow). H&E, hematoxylin and eosin.

CT-guided core needle biopsy revealed tumor fragments with partial translucent and opaque white flecks and pink areas in a rubbery to firm consistency. Histopathological examination showed dense collagenous matrix scattered with areas of psammomatous calcifications, nests of plasma cells, few mast cells, eosinophils, and lymphocytes (Figures 3, 4). No atypia or mitotic activity was seen. Immunohistochemistry showed positive expression of cluster of differentiation (CD) 34, B-cell lymphoma 2 (Bcl-2), and patchy staining for CD 99. It was negative for factor XIII a, smooth muscle actin, myosin, desmin, CD 117, anaplastic lymphoma kinase 1 (ALK-1), S-100, cytokeratin (CK) 8/18, or beta-catenin. The proliferative index (Ki-67) was less than 1%. These findings were consistent with CFT. Oncological and thoracic surgery consultations were obtained. Given persistent pain, a large size, the possible involvement of lung causing obstructive symptoms due to compression, and anticipated tumor progression, surgical resection was planned.

**Figure 5:**
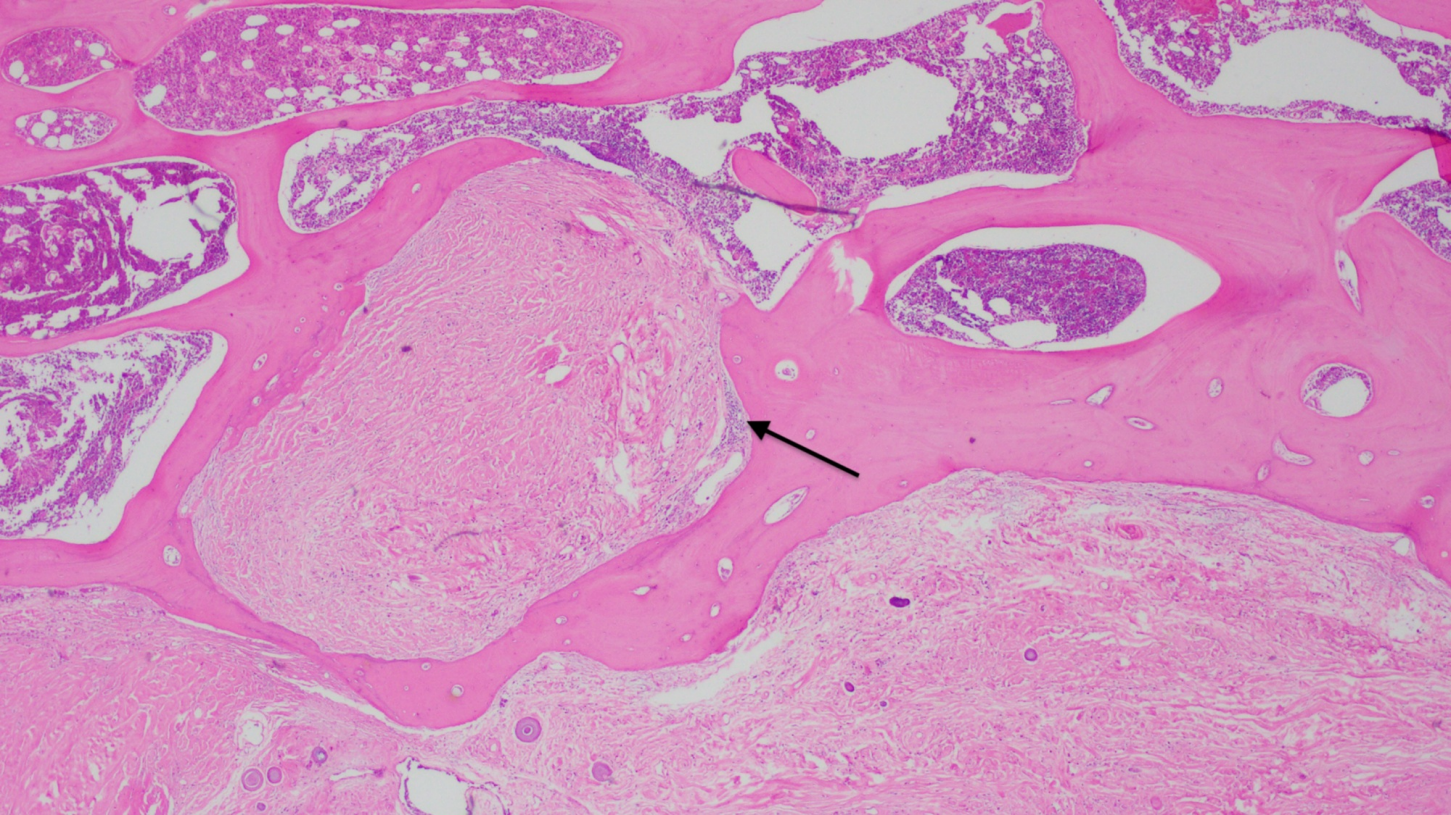
Tumor foci with one of the fragments (arrow) seen inside bone intermixed with normal rib histological structures. No invasion is seen.

The patient underwent thoracotomy. The tumor was firmly attached to the medial aspect of the aforementioned ribs in close proximity to the vertebral transverse process, almost completely decompressing adjoining lung. A 15 x 15 x 10 cm lobulated unencapsulated mass with a smooth, hard white surface was removed along with wedge resection of the involved portion of right upper and lower lobes and ribs 5 through 8 followed by chest wall reconstruction. Microscopically, the tumor was adherent to pulmonary tissue in an intimate relationship with ribs, but without local invasion. The benign tumor cells were notably intermingled with normal rib tissue structures; however, no evidence of cellular invasion was seen (Figure 5). Immunohistochemistry of the piecemeal mass confirmed the above findings, with CD 138 positivity in the collection of plasma cells. Postoperatively, the patient’s pain improved; however, he developed hypoxia secondary to recurrent right-sided pleural effusions. Pleural fluid studies revealed a nucleated cell count of 102,880/μL, with 100% neutrophils, 20,000/μL red blood cells, and two out of three positive light’s criteria (lactate dehydrogenase 2,788 U/L and a fluid protein [4.8 g/dL] to serum protein [7.7 g/dL] ratio greater than 0.5) with positive cultures for methicillin-sensitive Staphylococcus aureus.

**Figure 6:**
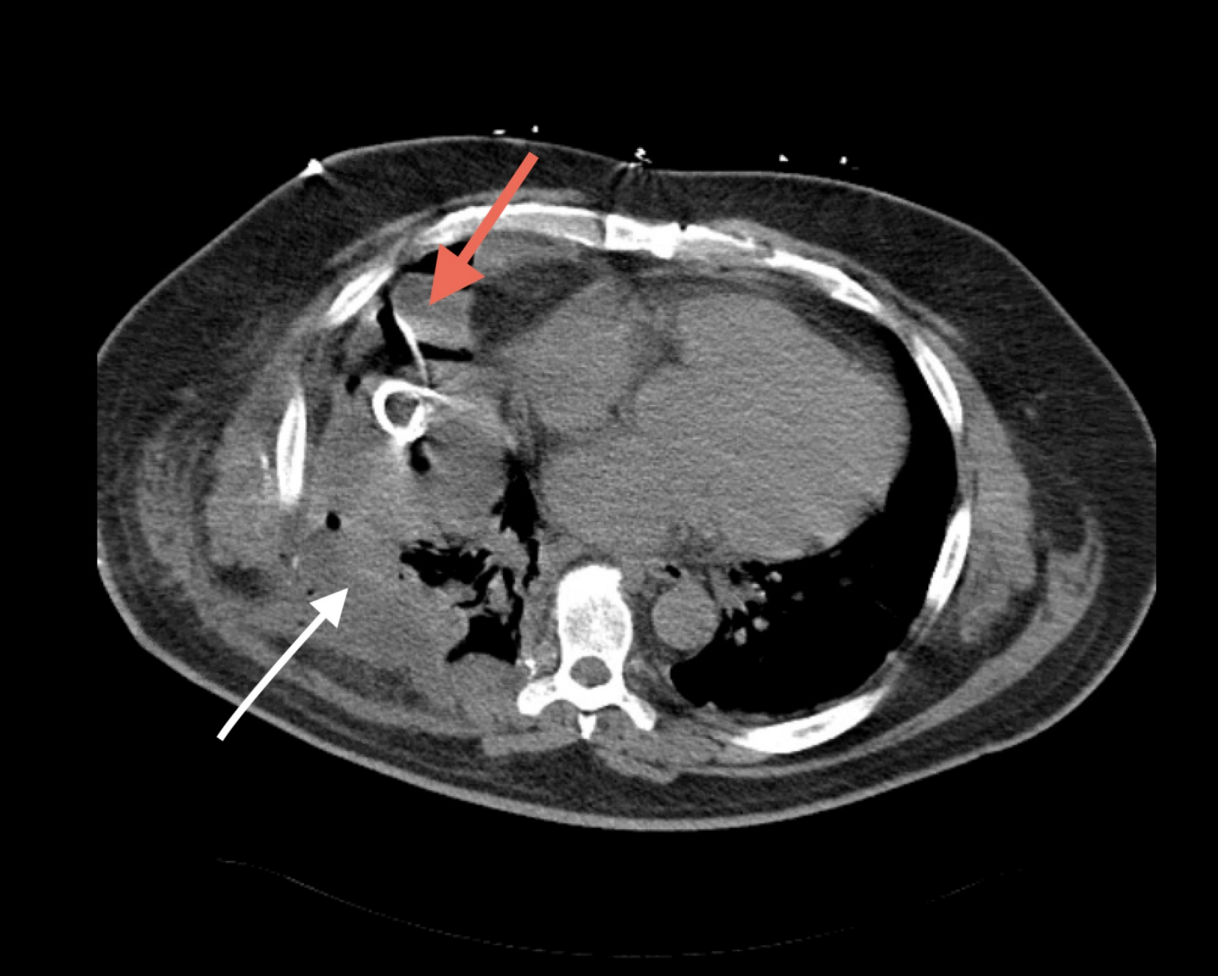
CT of the chest with a dominant fluid collection in the anterior aspect of the right chest (red arrow). There is also a loculated collection in the posterior aspect, with multiple septae (white arrow).

CT chest showed a large empyema in the right lower lobe requiring multiple invasive procedures for intercostal drainage (Figure 6). The patient was started on a six-week course of antibiotics and was discharged with improvement in pleural effusions. Over one year of follow-up, he did not show any signs of recurrence of the tumor. 

## Discussion

Due to its rarity, no randomized controlled trials exist to understand CFT. The limited evidence of its clinical presentation and pathogenesis comes from a few case reports and systematic reviews available in the medical literature. Owing to the abundance of inflammatory cells reported almost universally on histopathological examination, it was previously thought to be the result of a reactive process, representing end-stage inflammatory myofibroblastic tumor. However, later clinicopathological studies showed that the relationship between these two tumors was weak [3]. Consequently, in 2015 the World Health Organization (WHO) renamed this disease as ‘calcifying fibrous tumor’ due to its potential for recurrence and multifocal involvement in different organs, classifying it amongst the soft tissue tumors. A recent study on three patients with CFT of the pleura used whole-exome sequencing and identified the loss of a number of copies on chromosomes 6 and 8, hypothesizing its tumorigenesis [4]. It is now presumed to harbor specific gene mutations along with an ability to metastasize.

According to the largest systematic review which studied 157 cases of CFT, this tumor presents in a bimodal age distribution with one peak at 0-4 years and another between 25 and 34 years with a mild female preponderance [1]. Most patients are asymptomatic at diagnosis. When symptoms are present, they tend to be site-specific and can include chest discomfort, pain, dyspnea, and sometimes cough when arising in the chest wall cavity or wheezing, as in our patient, secondary to compression of airways [5-8].

Through this case, our aim is to increase awareness about this rare disease which should be kept in the differential diagnosis while evaluating lung masses. Although radiologically such large masses may appear highly suspicious for malignancy, biopsy is needed to prove their benign nature. Generally being non-aggressive, it does not invade the neighboring tissues, even while growing in size. Some cases have described entrapment of nerve bundles noted on microscopic examination, however without signs of invasion, once again suggesting its benign nature [9,10]. Notably, when arising from the pleura, it usually does not involve the lung parenchyma. Although our patient’s mass appeared to be invading the adjacent lung and adjoining ribs on imaging, this was ruled out on microscopic examination (Figure 5).

Prompt treatment is required and might involve surgical excision, with laparoscopic approach employed for smaller lesions. It is important to highlight that to our knowledge, our patient’s mass was the largest among reported CFTs in the literature and required extensive chest wall reconstruction including resection of the involved lung parenchyma. This led to complications like empyema, requiring additional invasive procedures, and further complicating the postoperative course of our patient. This underscores the importance of early diagnosis and treatment. Although difficult due to its asymptomatic presentation and incidental diagnosis in most cases, it is quintessential to prevent the sequela of rapid tumor growth and resulting compression of adjacent organs. Treating physicians should be cautious of these potentially life-threatening complications to provide efficient postoperative management and ensure patient safety. Recurrence is rare, with only a few reports documenting it on long-term follow-up [11].

## Conclusions

CFT is a rare entity that should be kept in the differential diagnosis of lung masses. It is important for medical professionals to be aware of its clinical presentation and course to allow early recognition and prompt treatment. Although generally benign, it can have deleterious effects when grown to exponential sizes, leading to aggressive surgeries and related post-operative complications, which can be avoided if caught early. Thus, providing high-value care for patients.
